# Similarity and economy of scale in urban transportation networks and optimal transport-based infrastructures

**DOI:** 10.1038/s41467-024-52313-6

**Published:** 2024-09-12

**Authors:** Daniela Leite, Caterina De Bacco

**Affiliations:** Max Planck Institute for Intelligent Systems, Cyber Valley, Tübingen, Germany

**Keywords:** Complex networks, Computer science, Civil engineering

## Abstract

Designing and optimizing the structure of urban transportation networks is a challenging task. In this study, we propose a method inspired by optimal transport theory and the principle of economy of scale that uses little information in input to generate structures that are similar to those of public transportation networks. Contrarily to standard approaches, it does not assume any initial backbone network infrastructure but rather extracts this directly from a continuous space using only a few origin and destination points, generating networks from scratch. Analyzing a set of urban train, tram and subway networks, we find a noteworthy degree of similarity in several of the studied cases between simulated and real infrastructures. By tuning one parameter, our method can simulate a range of different subway, tram and train networks that can be further used to suggest possible improvements in terms of relevant transportation properties. Outputs of our algorithm provide naturally a principled quantitative measure of similarity between two networks that can be used to automatize the selection of similar simulated networks.

## Abstract (web summary)

Planning effective urban network infrastructure often involves optimization principles that use a backbone network as a starting point. The authors propose an approach based on optimal transport theory to simulate real urban rail networks structure without need of initial backbone knowledge.

## Introduction

Transportation networks are a fundamental part of a city’s infrastructure. Their design impacts the efficiency with which the system is operated, hence, they should follow optimal principles while being constrained by limitations like budget or physical obstacles. Existing approaches for studying the quality of network design often rely on the analysis of the topological network properties, and relate them to optimal features like transportation cost, efficiency or robustness. These analyses are usually made *a posteriori*, only once the network has been constructed, and thus only resulting properties can be analyzed^1,2^. A different approach is that of posing a priori a principled optimization setup, where one defines a cost function that a network should minimize under a set of constraints, and then searches for optimal solutions in terms of network topologies. Numerous studies have explored this approach in biological networks, transportation networks, etc^3,4^. However, most of these methods rely on an existing backbone of a network infrastructure that can be optimized in terms of traffic distribution^5,6^ but do not consider the possibility of building the network from scratch, starting from a limited set of nodes. Alternatively, as optimizing over all possible topologies is difficult, one can investigate only various simple shapes from a predetermined set of possible geometries^7–9^ or rely on heuristics^10,11^. In fact, proposed solutions for the network design problem (also known as transit design problem) often rely on heuristics that do not necessarily generate equilibrium solutions^12,13^. Alternatively, bilevel formulations account for the needs of both passengers and network manager in a hierarchical way, framing the passengers’ objective as a constraint on the network manager’s one^14,15^, but solving them is NP-hard^16^. Another approach is that of using models that mimic biological networks. For instance, ref. ^17^ presented a two-step agent-based model that replicates biologically-grown networks and proposes them as a template for urban design. Nevertheless, the lack of a principled metric to measure the similarities between an observed network and a simulated one poses a challenge for making this evaluation effective. In addition, most of these approaches extract optimal networks starting from an initial backbone infrastructure, which impacts the resulting topology. Instead, we take a different approach and generate a network from scratch, starting only from a few origins and destination points in a continuous space, identifying where nodes and edges should be located.

In this work, we show that urban transportation systems can exhibit underlying network topologies similar to those that follow optimality principles as defined in optimal transport theory. Specifically, we propose a model to characterize real transportation networks based on a simple optimal transport framework, similar to what is observed in biological systems like the slime mold *Physarum polycephalum*, which adapts its network structure to reach food patches in an optimal way. A previous study by ref. ^18^ shows how this mold forms networks with comparable optimal transportation properties, e.g., efficiency and cost, to those of the Tokyo rail system, but provided no rigorous quantitative definition of network similarity beyond measuring these properties. We empirically validate our approach with a systematic characterization of the structure of several urban transportation networks and propose a rigorous definition of similarity in terms of optimal transport theory.

Urban transportation networks often exhibit different network structures based on the goals of network designers. For instance, some networks focus on connecting people living in the outer layers of the city to the city core, while others prefer to develop a robust infrastructure servicing the core^19^. Several studies have focused on analyzing properties like scaling laws and network connectivity^20–22^, which are indications of an underlying optimality mechanism that these networks might follow to make a city efficient, both in reduced infrastructure costs per capita and in increased productivity. However, our understanding of what optimality principles are captured in real transportation networks is incomplete. In fact, studying network properties could only partially explain the underlying mechanisms regulating network design, as each property captures a different aspect. Here, we take a different approach and build the network from scratch while comparing it with the real ones observed from data, starting with only a few shared nodes in input. Specifically, we model network structures observed in urban transportation networks by adapting a classical optimal transport framework to simulate a network-design problem dependent on realistic travel demand settings and using little information in input. We then compare the resulting networks with those observed from real data and assess their similarity. Importantly, the model can simulate different optimal strategies by tuning a parameter *β*, which interpolates between minimizing infrastructural and operating costs, in a similar fashion as in the principle of economy of scale, a fundamental concept in economy that establishes the relationship between growth and production costs. This principle affirms that, as the quantity of produced units rises, the average cost per unit of production declines^23^. This impacts the balance between the costs of producing and that of maintaining and operating the network. On one hand, this allows to simulate optimal networks that resemble those observed in real transportation systems more closely, as we tune *β*. On the other hand, by comparing the networks resulting for various values of *β* with those observed from real data, we can also assess the impact of the two types of cost in the design of various urban infrastructures.

We use this model to analyze several transportation networks from 18 cities and a national rail network^24–26^. Despite the complex nature of the mechanisms driving the design of transportation networks, we observe that multiple of the studied urban transportation networks follow a surprisingly similar topological pattern, as noticed in biological systems. We observed that in several cases, the optimal networks obtained with our approach have similar cost and performance to those observed in real ones.

## Results

### Modeling network design in transportation networks

**Fig. 1:**
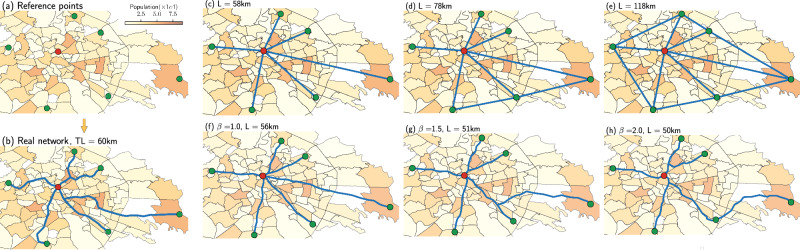
Problem setup for the subway network of Rome. **a** Given a set of real latitude-longitude coordinates denoting origins (green) and destinations (red), we aim to build a network structure that resembles well the observed public transportation network connecting those points, as in (**b**). **c**–**e** Intuitive ways to build a network structure by connecting origins and destinations, versus networks extracted with our optimal transport-based method in **f**–**h**. The only known information is the set of six origins (O) and one destination (D). We capture different optimization mechanisms by tuning the *β* parameter: in (**f**), the network is the shortest path-like structure, while in (**g**) and (**h**) we show examples of branched transportation schemes. This information is added to the population density across multiple urban areas (2019)^60^, where darker (lighter) colors indicate higher (lower) densities. *L* denotes the total length of the network, measured in kilometers.

Consider an urban area where a set of points of interest (POI) are located in certain positions in space. These may correspond to a combination of central and peripheral points where people work and live. The goal is to connect them by building a transportation network under the perspectives of an optimality criterion, based on the minimization of a cost-based energy functional. At this point, we do not observe any network but are rather free to use the whole space where the urban area is located, i.e., a 2D surface. From this, we need to select a set of points and edges connecting them, in other words, a network. In Fig. 1a, b we illustrate the problem setup for the subway network in Rome, where green and red markers denote an example set of such reference points and the lines denote edges in the observed metro network infrastructure. In the same figure, we show how existing stations are placed across urban areas with different population densities, as the evolution of subway networks often reflects the evolution of population and activity densities^27^.

In general, there are many choices for designing the network. For instance, in Fig. 1c–e we show three examples of intuitive shortest-path-like minimization solutions for the settings shown in Fig. 1a. These are however quite different from the observed network in Fig. 1b. The question we address is what network design principle is producing simulated networks that are more similar to those observed in real urban networks. Optimality could be defined in various ways depending on the network engineers’ and designers’ goals, but generally, it is not known what principles they used when building the network. Instead, we want to assess this by observing real data of transportation networks and fitting them with a flexible and computationally efficient optimization setup guided by optimal transport theory. In this context, a well-defined cost-based functional combines aspects that are critical for a transportation network: the cost of building the infrastructure and that of operating the network, in terms of power dissipation. This is relevant in scenarios where we expect infrastructures to be regulated by energy-saving requirements and the principle of economy of scale, where it is more convenient to consolidate traffic into fewer and larger edges. We expect this to be a reasonable assumption in urban transportation networks. As with any other natural or urban system, we do not know a priori what (if any) is the functional being optimized in the network under study. In fact, many of these systems (e.g., metro and tram) are built in phases^19^, where the design of an initial backbone structure is followed by several expansion steps, which may lead to suboptimal structures. However, our model allows considering, among the many possible choices, a simple but yet principled mechanism of optimality. By measuring the degree of similarity of networks that follow these principles with existing urban transportation networks, we can assess if the observed ones can be explained by this simple mechanism. And if not, we can point out alternative infrastructures that can be better in terms of certain relevant network properties, e.g., total path length. Our setting is simple because we consider only a limited input (few nodes that need to exist, e.g., main origin and destination stations), but otherwise do not consider any other constraint, besides main physical laws such as conservation of mass, and let the model select nodes and edges from a two-dimensional space where it can be optimal to drive passengers, tuning only one parameter. For this, we adopt the formalism recently developed by ref. ^28–30^ that generalizes to a continuous space the original idea of ref. ^31^. In particular, this allows starting with only a set of relatively few origin and destination nodes in input and then designing a network by exploring the 2D surface, i.e., without the need of an initial existing backbone. The idea is inspired by the behavior of the slime mold *P. polycephalum*, which dynamically builds a network-like body shape when foraging. One can thus consider a dynamics for the two main quantities involved, flows and conductivities, that implements this mechanism at any point in space. The stationary solution of this dynamics corresponds to the minimizer of a Lyapunov cost in a standard optimization setup, which has a nice interpretation in terms of infrastructure and operating transportation costs. From these solutions, one can then automatically extract optimal network structures using the approach presented in ref. ^32^. From now onwards, we refer to the algorithmic implementation of this approach as “Nextrout”.

Having introduced the main problem and ideas, we now briefly describe the model. Consider a surface in 2D and a set of points on it. Specifically, we denote a set of origins and destinations as *f* ^+^ and *f* ^−^, respectively. These contain the reference points where people enter and exit the transportation network. By defining *f* = *f* ^+^−*f* ^−^, mass conservation can be enforced with the constraint ∫ *fdx* = 0. The two main quantities of interest are denoted with *μ*(*x*, *t*), the transport density (or conductivity), and *u*(*x*, *t*) the transport potential. The former can be seen as a quantity proportional to the size of a network edge, while the latter determines the fluxes traveling along them. The dynamical equations in this continuous setting are

1 $$-\nabla \cdot (\mu (t,x)\nabla u(t,x))=f,$$

2 $$\frac{\partial \mu (t,x)}{\partial t}={\left(\mu (t,x)\nabla u(t,x)\right)}^{\beta }-\mu (t,x),$$

3 $$\mu (0,x)={\mu }_{0}(x) \, > \, 0.$$

Equation (1) determines the spatial balance of the flux, assumed to be governed by the Fick–Poiseuille flux as *q* = −*μ* ∇ *u*; Eq. (2) enforces optimal solutions, and represents the *P. polycephalum* dynamics in the continuous domain; Eq. (3) is the initial condition. The parameter *β* captures different optimization mechanisms: *β* < 1 enforces congested transportation, *β* = 1 is the shortest path-like and *β* > 1 is branched transportation. In Fig. 1f–h we show examples of different optimal configurations, with *β* = 1, *β* = 1.5 and *β* = 2.0. Here, we consider the cases 1 < *β* ≤ 2, where the approximate support of the conductivity *μ* displays a network-like structure. Under the lenses of a network, the conductivities can be viewed as the traffic capacities on the edges, hence Eq. (3) defines how the initial traffic capacities are distributed along the network, while Eq. (2) describes how these capacities evolve in response to the fluxes. As time evolves (i.e., ${\lim }_{t\to \infty }$), the equilibrium solution pair (μ^*^, u^*^) is reached. In refs. ^30,33^ the authors show that under certain assumptions, this equilibrium solution is a minimizer of the functional

4 $${{{\mathscr{L}}}}(\mu,u)=\frac{1}{2}\int\,\mu | \nabla u{| }^{2}dx+\int\,\frac{\beta }{2-\beta }{\mu }^{\frac{2-\beta }{\beta }}.$$

This can be interpreted as the network transportation cost, where the first term is a network operating cost (or power dissipation, it is the Dirichlet energy to the solution of the first partial differential equation), while the second is a non-linear cost to build the infrastructure. When *β* > 1, this second term corresponds to a principle of economy of scale, where it is more convenient to consolidate traffic into fewer (but larger) edges. This is the scenario we consider here. By changing *β*, one can tune their relative contribution to the total transportation cost, thus tuning the impact of the principle of economy of scale and how much concentrated path trajectories are. Besides being relevant for urban transportation, this strategy seems to be a fundamental mechanism in various natural systems, e.g., tree branches and roots, blood vessels or river networks^34,35^.

Alternative approaches can be considered to design a network infrastructure from simple mechanisms. Examples are cost-benefit analysis^36^, maximizing for efficiency^37^ accounting for paths and flows of passengers, or minimizing the total length, as in the Euclidean minimum spanning tree problem. In discrete settings, when an initial network backbone is given, the cost in Eq. (4) has been shown to be implicitly related to the total path length minimization accounting for the passengers’ trajectories^38^. One main difference between ours and these types of approaches is that we focus on a continuous space (as opposed to discrete settings) where the only necessary input is a set of origins and destinations, but otherwise no initial backbone network is given. This enables the design of a network from scratch, simulating where nodes and edges should be located in space to minimize the cost.

Once the optimal (μ^*^, u^*^) are obtained, one can then use the model described in ref. ^32^ to extract a final network structure, i.e., a set of nodes, a set of edges connecting them, and their weights proportional to the conductivities. This can then be compared with the one observed from real data and repeated for various values of *β*. It is important to remark that in our setting, besides the parameter *β*, the other input quantities that need to be specified are origins and destinations via the function *f*. By imposing non-zero entries to this function, a user automatically selects a set of nodes that will be necessarily present in the output network. Otherwise, no other set of nodes or edges needs to be given but is rather automatically learned by solving the optimization problem described above. This implies that similarity between simulated and observed networks trivially increases as we add more non-zero terms in *f*. Here we consider the non-trivial scenario where we fix only a small number of origins and destinations, as described in more detail below.

### Selecting origin and destination points

**Fig. 2:**
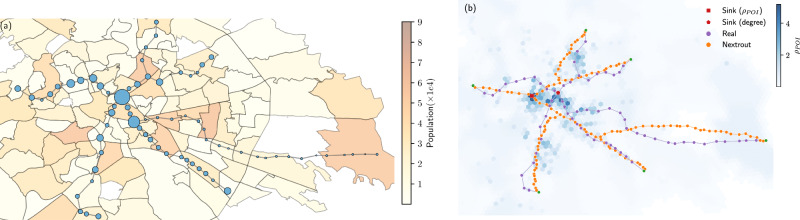
Comparing criteria to select origin and destination nodes. We compare two criteria to select the input nodes that we give to our algorithm, one based on a topological property (centrality) and one based on population and point-of-interest densities (*ρ*_POI_). **a** Stations with the highest and lowest annual traffic (2019), proportional to the node sizes, over the population distribution in Rome (in multiples of 10,000). The degree centrality of nodes in the observed is related to the traffic at stations. In particular, the central node with the highest degree (destination), has also the highest annual traffic. **b** Density accounting for population and distribution of points-of-interest (*ρ*_POI_), darker colors mean higher values, i.e., regions of higher relevance for transportation. When accounting for the Points of Interest (POI), we notice that the city center presents higher density (*ρ*_POI_) compared to the peripheral areas, therefore using centralities to select destinations is an approximation to real demands.

As we aim at extracting a network, our problem starts by defining a set of origins and destinations (O-D) points in the space with coordinates (*x*, *y*), where passengers might enter or exit. This choice necessarily impacts the output network, as the optimization problem depends on it. Ideally, one could reframe it by including O-D pairs as variables to be optimized along with conductivities and fluxes. But this becomes a different and more complex problem and it is not clear how to solve it. Here instead, we focus on the optimization setting introduced above and treat O-D pairs as fixed in input. While we limit selection to a small number of points, specifically one destination and few origins, it is important to decide where to place these input nodes in space. There are no universal criteria to define what are the most relevant points where city planners should add a stop to accommodate traffic when designing a network. However, existing infrastructures often evolve reflecting needs such as population increase or land usage^39^. With this in mind, we can assume that at least some of the existing nodes have already been placed in positions relevant to transportation needs. Hence, we select O-D pairs from important nodes as observed in existing urban networks. Specifically, we use centrality measures obtained from the original network: nodes with the smallest and highest centrality are assigned as origin and destination nodes, respectively. These measures might reflect the choice on where to place new stations that are often made by urban planners or transportation engineers, usually based on a variety of factors, such as population density, land use patterns or available funding^40^. For instance, in Fig. 2a we observe higher population density in peripheral regions, where the stations with lower centrality are located, whereas those with higher centrality are located towards the center, with lower population density. In the same figure, we show node sizes as proportional to the annual traffic in each station, as measured in 2019^41^. In this example, the highest traffic corresponds to the station with the highest degree centrality, thus reinforcing the choice of that node as a destination.

Another possibility is to incorporate urban features by using some measure of attractiveness, which takes into account the densities of POI and population in a given urban area, an approach also used in other works^37^. However, this strategy may not be scalable, as it requires the integration of several datasets, whereas using network centralities can be done automatically from the observed network data at no additional cost. We show an example of this for the metro network of Rome, to assess the extent to which these two strategies align. Here we categorize POIs into various types such as tourism, economy, culture, utilities, history, education, food, etc... as also done in previous studies^42^. We notice that the high centrality nodes found by the two criteria are located nearby geographically and yield similar simulated networks, see Fig. 2. Hence, we adopt the centrality criteria as a good approximation for attractiveness to select origins and destinations in all the networks investigated here.

### Investigating the similarity of optimal simulated networks and the observed transportation systems

We apply the proposed dynamics to empirical data collected from 18 different cities in multiple geographical regions around the world. For each city, we selected various available types of public transportation systems, such as rail, subway and tram, keeping the largest connected component. The networks considered in this manuscript have a few loops, as the dynamics can only retrieve loopless structures in the regime where network extraction is meaningful^32^. These networks could be seen as phase I in the classification of ref. ^19^, i.e., the initial phase where a backbone infrastructure is built, before a later evolution where further additional links are added through time. We expect these to be more likely to follow a global optimization criteria as the one formulated in our model (as opposed to other greedy heuristics for later extension phases). We thus measure the loop ratio *L*_*ratio*_ = *n*_*L*_/*E* as the number of loops divided by the number of edges and select networks with a low ratio, i.e., with *L*_*ratio*_ < 0.2 (see Methods for more details). One could in principle recover loopy structures by employing numerical schemes, e.g., superposition of different outputs^43^, but this is not the main focus of this work. Instead, we point towards directions on the loop recover perspective, presented later in this manuscript, by exploring an example of a more complex network structure as the New York subway.

**Fig. 3:**
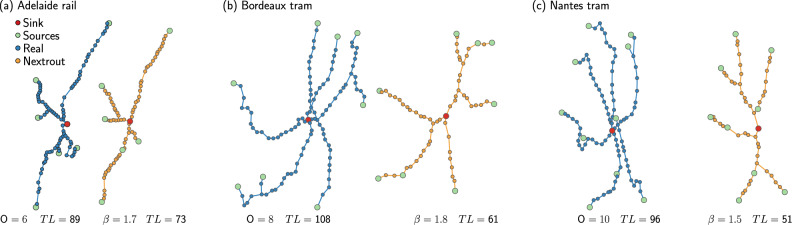
Example of different network topologies generated by Nextrout (yellow), plotted against the corresponding real network (blue). Green nodes represent those chosen as origins (O), whilst red nodes correspond to the destinations (*D*). All networks have *D* = 1. The TL measures the number of edges of each network. **a** Adelaide rail network, with *N* = 87 nodes. **b** Bordeaux tram network, with *N* = 108 nodes. **c** Nantes tram network with *N* = 97 nodes.

**Fig. 4:**
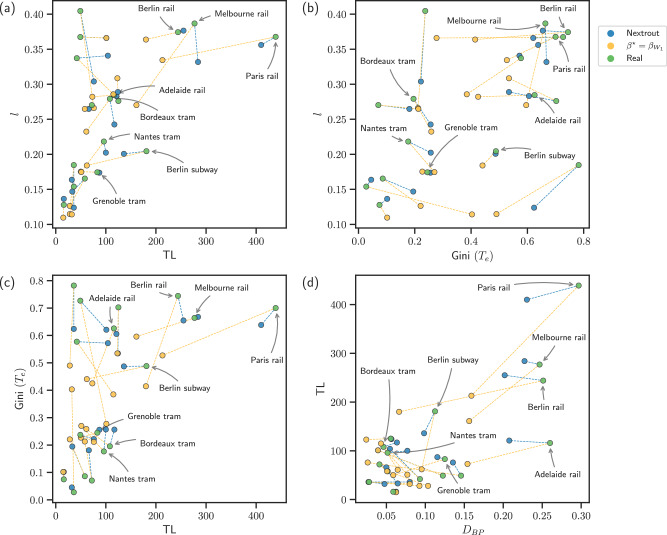
Performance measures for real and simulated networks. Real and simulated networks are distinguished by the green and blue colors, respectively, while yellow markers represent those selected by the Wasserstein similarity measure. The dashed blue lines are connecting the closest simulated networks with the real ones based on the given metrics, while the yellow dashes connect that given by the Wasserstein. **a** Cost (TL) measured in both simulated and real networks, plotted against the total path length. **b** Gini coefficient as a measure of traffic distribution, versus the total path length. **c** Traffic distribution in terms of the Cost. **d** Density of bifurcations plotted against the cost.

The applied Optimal Transport (OT) dynamics successfully describe the transportation network structures observed in different cities at a macroscopic level. While the selected transportation networks have different topologies and include multiple transportation modes, the networks reconstructed by Nextrout with only little information in input show a significant degree of similarity with the real ones (see Fig. 3a–c) for several of the studied cases, as evidenced by different similarity measures that we calculated to compare the topology of the simulated networks against the real ones, see Fig. 4 and next sections for details. This suggests the existence of simple universal optimality rules captured by the so-called Dynamic Monge-Kantorovich (DMK) dynamics for the modeling of urban transportation rail networks, similar to what has been observed for the behavior of the slime mold *P. polycephalum*.

**Fig. 5:**
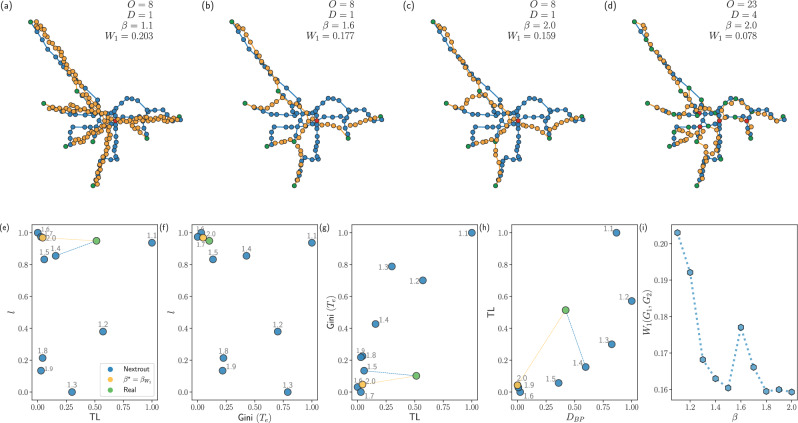
Wasserstein similarity measure between graphs for automatic selection of *β.* **a**–**c** We select the origin nodes based on those with the smallest degree, and a unique destination as the one with the highest degree. In (**d**) we show how the same network changes as we set more stations as initial input (O = 23 origins and D = 4 destinations), resulting in smaller Wasserstein, at the cost of a higher amount of information given in input. **e**–**h** We compare several network properties as measured in the observed and simulated networks. **e** The cost (TL) against the total path length (*l*), highlighting the different *β* for each obtained network. **f** Gini(*T*_*e*_) against *l*. The optimal network is equivalent to the one with minimal Wasserstein, i.e., *β* = 2.0. **g** TL against the Gini coefficient of traffic on edges (Gini(*T*_*e*_)). In this case, the optimal network corresponds to the one with *β* = 1.5. **h** TL against the density of branching nodes. The closest network in terms of the number of bifurcations for *β* = 1.4. **i** Wasserstein similarity measure for the simulated Grenoble tram networks as a function of *β*, in the setting of eight origins and one destination. The most similar network in terms of this measure is at *β* = 2, when *W*(*G*_1_, *G*_2_) is minimum. The peak at *β* = 1.6 is due to the absence of a few edges in the rightmost part of the network that results in disconnecting a small branch, thus causing the distance to increase.

An example of the different simulated networks is shown in Fig. 5a–d, where, as we increase the values of *β*, one can notice how the network infrastructure evolves from a shortest path-like (*β* = 1) to a branching topology, where two branches (*β* = 1.5) are created to lower the cost of building the infrastructure. In particular, the southern branch in Fig. 5b resembles an analogous one observed in the real subway network of Rome. As *β* increases to 2, this branch disappears to build a unique path that connects two destination points in the southeast side of the city, further lowering the cost to build the infrastructure. However, at this extreme value, the network is now less similar to the real one. Notice that in principle one can increase this similarity further, by simply adding more information in input in terms of origin and destinations (see Fig. 5d for an example with multiple origins and destinations). However, here we are interested in recovering macroscopic structure in a more challenging scenario where input information is strictly limited to one central destination and few peripheral origins.

Beyond a qualitative visual comparison, we explore how our simulated networks score in terms of core network properties relevant for transportation compared to the real networks. For this, we consider the cost, the total path length *l*, the distribution of traffic, and the density of branching points (or bifurcations; here we use the two terms interchangeably), for both extracted and original networks. Similar to ref. ^18^, we define the cost as the total length of the network (TL), i.e., the total number of edges. Passengers may not always take the shortest path, but may rather consolidate on fewer main arteries (e.g., to minimize the number of stops or connections)^44^, a behavior that can be captured by a DMK discrete dynamics (built-in the filtering step of Nextrout) by varying *β* and extracting the flows *u*_*e*_ on edges, quantities proportional to the number of passengers using an edge. Hence, we consider an alternative measure of total path length as $l: \!\!={\sum }_{e\in {E}_{i}}{{{{\rm{l}}}}}_{{{{\rm{e}}}}}| {u}_{e}|$, where l_e_ is the Euclidean distance. This takes into account *u*_*e*_, the flow of passengers on an edge *e*, and its absolute value ∣*u*_*e*_∣ is proportional to the number of passengers traveling on an edge *e*, i.e., how traffic is distributed, assuming that passengers follow optimality principles to consolidate paths. This is a reasonable assumption in rail networks as the ones studied here, where the cost to build the infrastructure can be high (and thus should be minimized) and minimizing traffic congestion is not as relevant as in, e.g., road networks. In our experiments, we extract optimal flows *u*_*e*_ by running the discrete DMK dynamics on the extracted and real networks, using the same sets of origins and destinations as used in the original network extraction problem, setting *β* = 1.5. This information can also be used to measure the macroscopic behavior of traffic on edges, which can be measured using the Gini coefficient^45^ (Gini(*T*_*e*_)) on the traffic *T*_*e*_ = ∣*u*_*e*_∣. This coefficient ranges from [0, 1], where the closer to 1, the more unequal is the traffic distribution on the network. Finally, we calculate the percentage of bifurcations (*D*_*BP*_) as the fraction of nodes with degree equal to 3. In several cases, the simulated networks display similar properties as those observed on the real ones, as shown in Fig. 4. While similarity differs depending on the property and datasets vary in their range values, we notice that most of the datasets have at least a pair of properties that have a close value between simulated and observed networks. For instance, in Fig. 3a we notice that Adelaide rail has an intuitively similar path length, which is confirmed in Fig. 4a. Furthermore, the same network shows comparable results for traffic and cost. As for the tram networks of Bordeaux and Nantes in Fig. 3b, c, we observe similar behavior for cost and traffic.

### Automatic selection of similar simulated networks

Our method allows extracting various simulated networks by varying the parameter *β*. One can select the one that more closely resembles the observed one in terms of a particular metric of interest, as shown in the previous section. However, different metrics may lead to different most similar simulated networks (i.e., different *β*), which may not be ideal for a practitioner willing to consider an individual simulated network that resembles well the observed one in terms of all metrics. Hence, the need for a principle automatic selection criteria for choosing the value of *β*.

The formalism introduced in the previous section suggests a natural way to tackle this problem by considering the Wasserstein similarity measure, a main quantity in optimal transport theory^46^. Given two graphs that need to be compared, intuitively, this measure captures the minimum “effort" required to move a certain distribution of mass from one to the other. Similar ideas based on optimal transport to measure similarity between graphs have also been proposed in recent works^47,48^. Here, we describe our proposal for a similarity measure and thus automatic selection of *β* in detail. Denote the observed network with *G*_1_(*V*_1_, *E*_1_) and the one obtained from the model introduced in previous sections with *G*_2_(*V*_2_, *E*_2_), where *V*_*i*_, *E*_*i*_ denote the set of nodes and edges, respectively, *i* = 1, 2. We consider the union graph *G*_*U*_(*V*_*U*_, *E*_*U*_), with sets of nodes *V*_*U*_ = *V*_1_ ∪ *V*_2_ and edges *E*_*U*_ = *E*_1_ ∪ *E*_2_. One can further assign weights *w*_*e*_ ∈ *W*_*U*_ to the edges *e* ∈ *E*, for instance using the Euclidean distance *ℓ*_*e*_ between the nodes *i*, *j* ∈ *V*_*U*_, where *e* = (*i*, *j*), or simply binary values {0, 1}. Notice that the observed network *G*_1_ may contain nodes that do not correspond exactly to nodes in *G*_2_, because in this continuous setting the model uses all the 2D space where the original network is embedded. Only the input origin and destination nodes are guaranteed to be present in both graphs, as they are given in input to the model.

Working on this union network, we then exploit a similar setting as the one already introduced with the model to obtain a Wasserstein-based similarity measure between *G*_1_ and *G*_2_. Specifically, we denote with *B* the unsigned incidence matrix of *G*_*U*_ with entries *B*_*ie*_ = +1 if node *i* is a start or end point of the edge *e* and 0 otherwise. Defining *q*_*i*_ as an indicator vector for the edges in *G*_*U*_ that are also in *G*_*i*_, i.e., *q*_*ie*_ = 1 if *e* ∈ *E*_*i*_, and *q*_*ie*_ = 0 otherwise,  ∀ *e* ∈ *E*_*U*_ and *i* = 1, 2, we can set the origin and destination vectors *f* = *f* ^+^−*f* ^−^, such that *f* ^+^ = *B* *q*_1_ and *f* ^−^ = *B* *q*_2_, so that *G*_1_ contributes to *f*^+^ and *G*_2_ to *f*^−^. By running a discrete dynamics analogous to the continuous one described in Eqs. (1) to (3), which can be done using Nextrout^32^, one naturally obtains our Wasserstein similarity measure defined as:

5 $${W}_{1}({G}_{1},{G}_{2})={\sum}_{e\in {E}_{U}}{w}_{e}\,{\mu }_{e}\,,$$

where *μ*_*e*_ are the optimal solutions for the conductivities on *G*_*U*_ and *w*_*e*_ is the weight of edge *e* = (*i*, *j*). Here, we fix this to be the Euclidean distance between nodes *i* and *j*. Examples of how the Wasserstein measure changes depending on the different output networks are shown in Fig. 5i, where we show simulated networks and report their Wasserstein measure from the observed network of the Grenoble tram (*N* = 80 nodes). Intuitively, the Wasserstein similarity captures how much “cost” is “paid” to move information between *G*_1_ and *G*_2_. This means that the more similar these networks are, the lower the Wasserstein is, i.e., when *G*_1_ = *G*_2_, *W*_1_ = 0, and if there are no nodes connecting them, *W*_1_ → *∞*. In Fig. 5b we show an example where *W*_1_ is higher simply because there is a disruption in one of the branches of the network, thus increasing the cost to move from the real network to *G*_2_ = *G*_*β*=1.6_. Notice that if similarity is chosen to be defined in terms of the cost, the closest network to the real one would be that with *β* = 1.4, as shown in Fig. 5e.

We further validate this measure by comparing it with other selection criteria based on the topological properties described above and found that the simulated graph selected with the Wasserstein measure has, on average, higher similarity with the real networks compared to the other selection criteria, across various properties. In other words, it shows transportation properties that are consistently more aligned to those behold by the observed network, see Supplementary Information S1.

### The New York subway system: a look into more complex structures

The New York subway is one of the largest and busiest transportation systems in the world. Due to its size and complexity, navigating through such network might be difficult for humans^49^, but understanding its properties and structure could be indicative of improvement to city planners and urban designers.

**Fig. 6:**
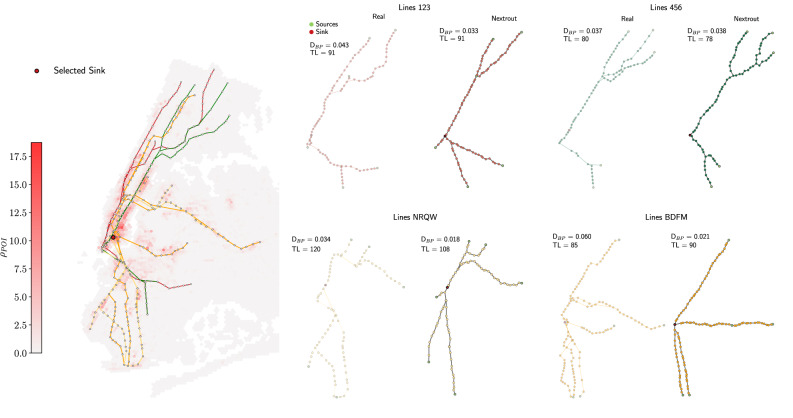
Comparison for real and simulated networks for major lines of the subway system in New York, with distribution of POI. We select the four major lines (left panel), here shown along with the distribution of the density of POI (density varies as in the colorbar). We report the cost (TL) and density of branching points (D_*BP*_) for both real and simulated networks. Here we set *β* = 1.1 for the orange line, *β* = 1.5 for both red and green lines, and *β* = 1.6 for the yellow line.

In the scope of recovering such a complex structure, the strategy of selecting only a small number of origins and destinations, as done for the studied networks so far, might produce networks that are far from similar to the real one, especially given the high complexity of this particular system (see Fig. S7). We thus use a different approach that could be a pointer towards recovering structures from major urban transportation systems. Each line that comprises the subway infrastructure of New York could be seen as an independent network itself. With this assumption, we selected four major lines (red, green, orange and yellow), extracting the nodes with lower degree as origins and one common destination for all of them, corresponding to the point with higher density of POI (see Fig. 6).

We notice that in terms of cost (TL), our simulated networks have similar values in all cases, with equal performance for the red line, and a small difference for all the other lines. For the density of branching points (*D*_*BP*_)—besides an intuitive visual similarity—we observe comparable results. For instance, the green line (456) has similar branches both on the north and south sides, and similar considerations apply to the red line (123) and the north side of the yellow line (NRQW). One can also investigate how main differences are distributed in the orange line (BDFM), where there is not much similarity between observed and simulated infrastructures. This is because two destinations on the east side are traversed by a unique branch in our simulated network, while the real one splits them into two branches. Furthermore, the southern part of the network also shows a distinct behavior, where our simulated network has two branches, the real one splits into a more complex pattern that cannot be explained by our optimality principles. While it is not clear whether these differences are due to different underlying optimization rules or a lack of optimality in the observed network, our method enables practitioners to identify key insights on principled alternative designs where optimality is clearly defined in terms of network operating and infrastructural costs.

### Initial network development: the French Railway in the 1850s

Our model builds a network backbone from scratch, with a global optimization that follows a principle of economy of scale. This could be particularly suited to study the initial development of a rail network infrastructure, as opposed to later stages where the network is gradually extended. We thus study a real scenario of the French railway system where we have access to historical information about network development in time^25^, focusing on an initial phase around the year 1850. At that stage, the network topology contains multiple connected components but no loops - which only appear in a later stage, around several years later (see Supplementary Information S6, Fig. S9).

**Fig. 7:**
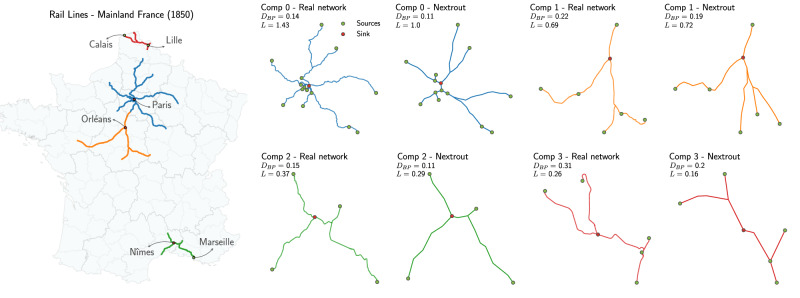
Simulating the initial French Railways in the year 1850. Left: the original observed network, with connected components represented in different colors. Right: the networks simulated with our model for each component, given a set of origins and one destination. On top we report the density of branching points *D*_*BP*_ and the total Euclidean length *L*. Origins and destinations have the same coordinates in all real and simulated networks.

We focus on the four biggest components with more interesting topologies, as the remaining components are either too small or simple straight lines, and select the node with the highest degree as a destination. In the biggest component, for instance, this corresponds to Paris. In Fig. 7 we show examples of real and simulated networks highlighting the *D*_*BP*_ and the total length (*L*), the latter measured given the longitude and latitude coordinates mapped into a [0, 1] system of coordinates. We note various degrees of similarity in the different components between observed and simulated network. For instance, the biggest component has similar *D*_*BP*_ but the observed network has a larger total length, mainly due to branches taking slightly longer detours to reach the sinks and the two small branches south-west of Paris being split from Paris onward into two in the observed network, while they are only later split in the simulated one. A similar behavior is observed in component 3 (north-est France), where we see the real network splitting earlier on than what simulated, thus causing a longer total length. The other two components have higher similarity in terms of both metrics, in particular, component 2 (south France) has a main branching point est of Nîmes similarly located in the observed and simulated network. The higher path length in this case is due to a longer detour of the southern branch. These types of detours could be caused by geographical obstacles that are not included in our more coarse-grained model.

Understanding the underlying optimization principles that drive how transportation networks changed and evolved over time might point towards creating better transportation systems. Our approach is a pointer towards comprehending the initial stages of such evolution, particularly suited for systems that follow the principle of economy of scale.

### Improving network properties

**Fig. 8:**
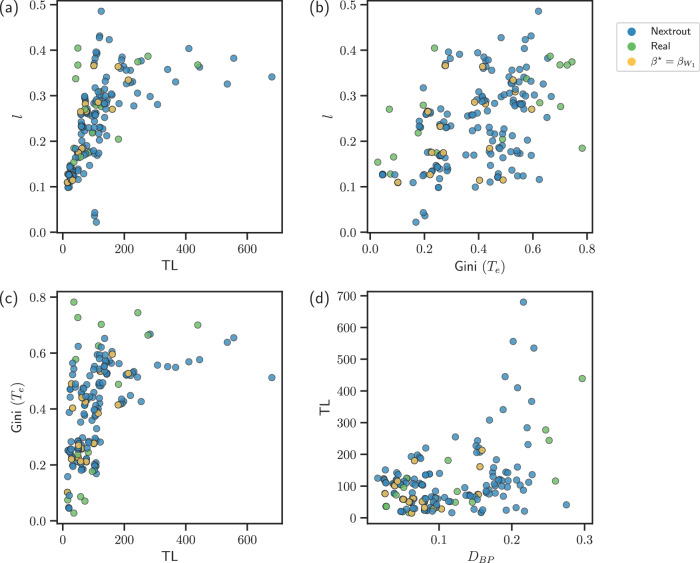
Comparison of simulated and observed networks. We show the values of the main transportation properties investigated in this work for real and simulated networks from (**a**)–(**d**). Simulated networks cover a wider range of properties' values, thus allowing in particular to select network that have lower or comparable values of these properties than those observed in the corresponding real networks.

Simulating networks that follow optimality principles and resemble well those observed in real datasets can be used to assess how urban transportation networks perform in terms of main transportation properties. This can guide network managers towards potential measures directed at improving certain properties. This possibility is conveniently enabled by our approach, as by continuously tuning the parameter *β* we can simulate various transportation scenarios, thus assessing how a network can increase or decrease a certain property. In Fig. 8 we show the main properties in all the networks simulated with our model and compare with those observed in real data, aiming to compare their trade-offs between various performance metrics. In general, we notice how simulated networks cover a wider range of values than the real ones for the four transportation properties investigated in this work. This allows obtaining, for instance, networks that have shorter total path length *ℓ* with a comparable cost, as shown in Fig. 8a where many simulated networks are located in the regime 0 < TL < 200 with *ℓ* sharply dropping towards 0.1, while many real networks have *ℓ* > 0.1. A similar behavior is observed also for the traffic against TL in Fig. 8c, where simulated networks cover areas of the plot where traffic is less congested (smaller Gini(*T*_*e*_)), in contrast to several real networks. Among the simulated networks, those selected according to the best Wasserstein measure tend to have lower cost and a smaller percentage of bifurcations *D*_*BL*_, indicating that this measure encourages not only the usage of a lower amount of edges but also nodes with low degree.

## Discussion

Planning transportation systems in a city is a challenging task. This study has shown that some urban transportation systems with a small number of loops can be simulated by simple principles based on optimal transport theory and economy of scale. Using empirical data from one national railway and multiple rail transportation types across 18 cities, our model provides simulated networks obtained with little information in input and no a priori backbone network structure, exhibiting properties that resemble those observed in real transportation systems to various extents. Our model interpolates between various transportation regimes by tuning a single parameter, while allowing for a natural definition of a similarity measure to compare the simulated networks with those observed in real systems. We observed how the selected networks with this criterion can exhibit transportation properties that on average resemble the corresponding real networks well or point towards alternative infrastructures that improve relevant topological properties.

A limitation of this study is that our OT-based model does not capture infrastructures with loops, thus limiting its applicability to rail networks, or subway and rail networks with a very small density of loops. Possible extensions of the formalism presented in this work to account for loops are an interesting direction for future work. Besides simple heuristics and beyond the example we presented for the New York subway, one can make other interesting modeling choices to effectively tackle this problem. For instance, one could generalize our approach to situations where travel demands are treated stochastically^50–52^ or change in time^53^, scenarios where in certain regimes an OT-based approach can naturally lead to the formation of loops. Similarly, loops could emerge in multicommodity settings where fluxes of passengers are distinguished by their origin and destination stations, using an OT-based multicommodity framework as the one proposed in refs. ^38,54,55^. Both directions, provided they could be generalized to a continuous setting as the one studied here, can potentially result in optimal simulated network infrastructures capturing properties that differ from the ones analyzed here, e.g., robustness to disruptions.

Another limitation is the assumption that networks are static, i.e., do not change in time. It would be interesting to compare how differences between simulated and observed networks may arise because different network branches may have been developed in different time periods. This could be used to study the evolution of transportation properties in time, as done in refs. ^27,56^. Similarly, the networks studied in this work are often one layer of a multimode network. Integrating other transportation modes into a multilayer formalism and suitably adapting our OT-based approach, e.g., borrowing ideas from^57^, could give us a deeper understanding of optimal network design in interconnected urban systems.

Our model takes a few inputs (origins and destinations), but it does not consider any geographical obstacle. This can result in fine-grained mismatches between simulated and observed topologies due to longer detours in the real infrastructures to avoid these physical obstacles. In principle, this could be incorporated into our model by properly adding extra terms in the dynamical equations that drive flows and conductivities differently based on the location in space. However, this would require fine-grain details to be specified in input, an information that may not be easily available.

In our work, we focus on designing a network from scratch. This is relevant in cases where infrastructures are relatively new or did not change considerably compared to their initial design, as in the cases we investigated here. However, this may be limited to studying infrastructures that have evolved over time. For these scenarios, it would be appropriate to consider how our model can be adapted to study network growth, where an initial backbone is further extended with new branches. This problem is related to the interplay between an urban transportation network and the distribution of its underlying population, as there could be a co-evolution between the two that should be taken into account^58^.

In summary, there are many factors contributing to the development of urban transportation networks. Our simple optimization scheme provides a principled and computationally efficient benchmark for comparison with real-world networks. By interpolating between different transportation regimes, we can vary the degree of similarity between the networks simulated by optimal transport principles and those observed in real systems. In particular, measuring relevant topological properties on simulated network resulting from different parameters’ values against those observed in real networks can give us indications on how to improve transportation performance when taking into account principles of optimal transport and economy of scale.

## Methods

### Data collection and analysis

We collected network data from various public transportation networks from 18 different cities^24^ and one national railway^25^. Network statistics are detailed in Table 1. Each city had one or multiple transportation modes available. Node ids were associated with the longitude and latitude coordinates of real stations for multiple means of transportation, as well as possible connections between them. Our main goal was to analyze each network individually, therefore we did not address the multilayer case where connections among the different means of transportation exist. For instance, if rail and subway stops have the same coordinates, they are treated as distinct in each network.

**Table 1:** Description of real networks considered

City	Transport mode	*N*	*E*	# Comp	*L*_*ratio*_	*O*	${D}_{\{{d}_{i},{B}_{i}\}}$
Adelaide	rail	88	116	1	0.25	6	1
Berlin	rail	203	258	1	0.21	19	1
Berlin	subway	169	181	1	0.072	14	1
Bordeaux	tram	110	110	1	0.009	8	1
Brisbane	rail	297	367	1	0.193	10	1
Dublin	rail	59	73	1	0.03	10	1
Grenoble	tram	80	83	1	0.048	8	1
Helsinki	subway	17	16	1	0.0	4	1
Lisbon	rail	48	49	2	0.041	9	1
Luxembourg	rail	43	56	1	0.025	11	1
Melbourne	rail	219	290	1	0.248	22	1
Nantes	tram	97	96	1	0.0	10	1
New York (full)	subway	423	506	1	0.17	22	1
Paris	rail	337	445	1	0.244	24	1
Prague	subway	24	23	3	0.0	6	1
Rome	subway	73	72	1	0.0	6	1
Toulouse	subway	37	36	1	0.0	4	1
Venice	tram	37	38	1	0.053	4	1

We report the main network statistics as number of nodes *N*, number of edges *E*, number of components # Comp, number of origins *O*, of destinations *D*, and loops ratio *L*_*ratio*_ defined as the number of loops divided by the number of edges, and selecting networks with *L*_*ratio*_ < 0.2, as higher values would require the recovery of loops in the extracted networks.

To avoid possible redundancies or inconsistencies in the data, such as duplicated nodes or edges that looked too long, we performed a preprocessing step. Specifically, we considered a threshold *τ* that corresponds to the minimum distance in kilometers between the pairs of nodes that had the same node ids and no connections between them, matching features stored in the node metadata such as names of the real stations. If the Euclidean distance *d*(*i*, *j*) between nodes *i* and *j* was smaller than this threshold, we collapsed the two nodes into one, i.e., *i* = *j*. This was used to avoid those entries and exits of each station would be counted as two distinct nodes in the same network, and possibly affecting the selection of origins and destinations.

To match the latitude and longitude coordinates of the datasets with those in the 2-dimensional plane that Nextrout uses to solve the continuous problem, we re-scaled every pair (lon, lat) to a (0, 1) system of coordinates. Starting with a total of 64 data points (networks), we extracted the individual disconnected components and the number of loops for each of them. Network extraction was performed on the biggest components only.

We extract networks using Nextrout^32^, selecting 1 < *β* ≤ 2 such that for every pair (*origins*, *destinations*) we simulate 10 different networks. Since the extracted networks may contain redundancies, we remove them using the graph filtering step from Nextrout. Outputs of this step have less redundant structures and are closer to the optimal topologies.

#### Selecting origins and destinations with points of interest

For the 16 studied networks, we used origins and destinations based on the degree and betweenness centrality measures. The degree centrality *d*_*i*_ of a given node is defined as the number of edges connected to it. The betweenness centrality is defined as the frequency with which a node is on the shortest path between all other nodes,

$${B}_{i}={\sum}_{i\ne j\ne k}\frac{{\sigma }_{ik}(\;j)}{{\sigma }_{ik}},$$

where *σ*_*ik*_ is the total number of shortest paths from node *i* to node *k* and *σ*_*ik*_(*j*) those shortest paths passing through *j*.

Terminals were chosen as follows: nodes with *d*_*i* _≤ 1 are assigned as origins, while those with ${d}_{i}={\max }_{n}\left\{{d}_{n}\right\}$ or ${B}_{i}={\max }_{n}\left\{{B}_{n}\right\}$ as destinations. We selected this set {origins,destinations} to be small, in order to use the least amount of information in input. In multiple cases the set of origins was equivalent in both centralities, with the difference being on the location of the destinations, thus the output networks were different. In terms of final networks properties, the results are comparable for both studied centralities (see SI for more details).

In order to account for other measures of attractiveness for the destinations and investigate the impact of including realistic information about urban areas, we explored a different approach for the cities of Rome and New York, by looking at a measure that accounts for both population distribution and land usage. To do that, we collected both population and distribution of POI from Open Street Map (OSM) data and mapped them to an H3 tiling discretization of the space. We then defined the density of POI as *ρ*_POI_ = *P*_*ij*_*W*_*ij*_, where *P*_*ij*_ is the population density and *W*_*ij*_ is the number of POI for the corresponding *ij* cell. Our hypothesis is that stations with higher centralities correspond to higher density cells. The destinationis then assigned based on the center of this H3 cell with highest *ρ*_POI_. In Fig. 2b we show how this new criterion generates a configuration that leads to a different optimal network but still preserves similarity compared with the real network. We also notice that the highest centrality node is connected to regions with higher densities compared to the peripheral areas, where the origins are placed.

## Supplementary information

Supplementary Information

## Data Availability

Due to its high volume, the synthetic data generated in this study can be obtained from the corresponding author upon request. An example for the generated networks of the Rome subway is available at ref. ^59^.

## Code availability

The open source codes and executables are available at ref. ^59^.
