# Supplementary material for: Similarity and economy of scale in urban transportation networks and optimal transport-based infrastructures
Source: Nat Commun. 2024 Sep 12;15:7981. doi: 10.1038/s41467-024-52313-6 (PMC11393328; doi:10.1038/s41467-024-52313-6)
Supplement: Supplementary file 1 — Supplementary Information [file 41467_2024_52313_MOESM1_ESM.pdf]

Supplementary information for: “Similarity and economy of scale in urban transportation networks and optimal transport-based infrastructures”

Daniela Leite[1,*] **and Caterina De Bacco**[1,*]

[1]Max Planck Institute for Intelligent Systems, Cyber Valley, 72076, Tübingen, Germany
[*]caterina.debacco@tuebingen.mpg.de

Supplementary note 1: validating the Wasserstein metric

To evaluate how the simulated networks selected with our Wasserstein-based metric perform in terms of the main topological properties defined along the main text, we define a similarity ratio r_p for each of them. Given a particular property p, we compute the ratio between the value measured on both simulated and observed networks. Thus, when $r_p = 1$, the simulated network extracted by Nextrout matches perfectly with the observed one in terms of property p. For instance, when p is the cost, then $r_{TL} = TL_{\text{Nextrout}}/TL_{\text{real}}$.

In Figures S1 and S2 we show the ratio values for the multiple topological properties, comparing them across different automatic selections of β. Specifically, we obtain for each property p the value of β_p that leads to a simulated network closer to the observed one in terms of property p, i.e. corresponding to a r_p closer to 1. By definition, the simulated graph obtained with β_p has the best r_p for property p, but this may not be true for other properties measured on that same graph. For instance, the graph selected with β_{TL} (second row of Fig. S1) as $r_{TL} \approx 1$, thus reproducing well the number of edges, but it tends to largely overestimate both $Gini(T_e)$ and the number of bifurcations, with $r_p > 2$ for these two properties (see Fig. S2). Instead, we observe that the simulated graph selected with the Wasserstein measure (first row of Fig. S1) has on average r_p closer to 1 across various properties. In other words, it shows transportation properties that are consistently more aligned to those behold by the observed network.

To validate the consistency of the Wasserstein measure, we compute how each data point in Figures S1 and S2 differs from the perfect match $r_p = 1$. Specifically, we define the minimum mean displacement $\mathscr{D}_d = \frac{1}{|p|}\sum_p \left(1/n \sum_{i=1}^{n} |r_{p_i} - 1|^d\right)^{\frac{1}{d}}$, where p refers to each considered metric ratio ($|p| = 4$), $n = 17$ is the number of data points and $d = 1, 2$. The closer to 0, the less the considered measure deviates from the perfect score. We found that the Wasserstein measure deviates with $\mathscr{D}_{d=1} = 0.5$, whilst the cost has $\mathscr{D}_{d=1} = 0.61$, the total length $\mathscr{D}_{d=1} = 0.69$, the traffic $\mathscr{D}_{d=1} = 0.35$ and the density of bifurcation points has $\mathscr{D}_{d=1} = 0.32$. Similar results are found with the square displacement $d = 2$. The traffic and number of bifurcations have the lowest displacements, with the Wasserstein following. As the Wasserstein tends to select networks with higher β (not shown here), this measure encourages smaller cost (TL). The fact that we see a higher displacement than the one of traffic and density of bifurcations is a sign that real networks could be further optimized in terms of TL. Nevertheless, the gap is small and beyond problem-specific properties (as the traffic or density of bifurcations). These results confirm the benefit of using our Wasserstein-based similarity measure to automatically select a simulated network across different values of β in our Optimal Transport-based setting. More generally, it provides a robust and meaningful measure to compare network structures that can be used in applications beyond the one considered in this work.

As the final optimal network changes depending on specific selected transportation properties, a natural question is how distinct these networks are and how do such properties favor different optimal β. In Fig. S3 we show an example for the subway network of Berlin to illustrate this difference. While $\beta^\star = \beta_{Gini}$ favors a network with more branches and wider coverage, for $\beta^\star = \beta_{D_{BP}}$ the optimal network is that with fewer branches. This difference is thus evaluated across multiple properties using the metric ratios. Overall, we notice that the Wasserstein encourages smaller path length, but higher traffic and density of bifurcation points (which is confirmed by the minimum mean displacement).

Supplementary note 2: Wasserstein weights

The Wasserstein similarity measure (W_1) is designed to capture the amount of ‘effort’ necessary to move information between the original network and the one extracted using our pipeline. Since it is defined as the weighted sum of the optimal fluxes, the choice of such weights might influence the final optimal network. We compared the differences of selecting unitary or euclidean weights in Fig. S4 (b).

(a) $\beta^{\star} = \beta_{Gini} = 1.5$ (b) $\beta^{\star} = \beta_{W_1} = 1.7$ (c) $\beta^{\star} = \beta_{D_{BP}} = 2.0$

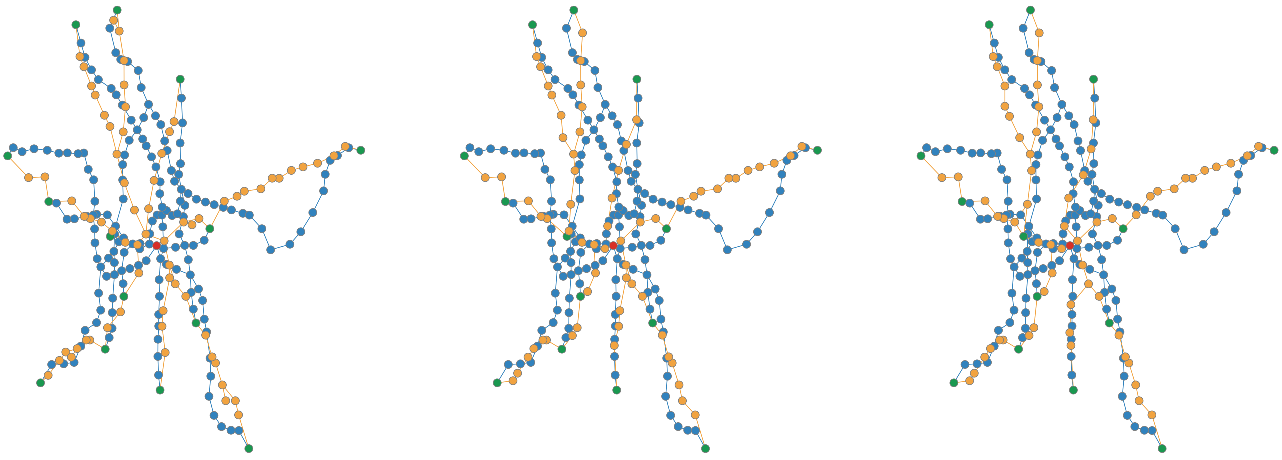

Figure S1. Counts for the metric ratios of the Wasserstein and cost (TL). Given a particular metric (e.g. Wasserstein, first row), we select the optimal β for each data point and measure the ratio across the multiple considered metrics. The red lines highlight the average for the optimal β given by the Wasserstein measure. We then repeat this procedure for the TL and highlight how the average of $\beta^{\star} = \beta_{Wass}$ changes given other optimal β.

Supplementary note 3: Centrality criteria for selecting origins and destinations

The first step of our pipeline consists in defining the set of Origin-Destination pairs (OD) taken from a real network. We perform a preprocessing step to remove possible redundancies found in the original spatial data collected from[1].

We map each stop, which consists in a pair longitude-latitude, to a node in a $[0,1]$ system of coordinates as a starting point. We then perform the preprocessing to obtain the original network mapping, and compute different network centralities to define the set of OD points: nodes with lowest values of degree, betweenness and closenness centrality are set to be origins and those with the highest values of these properties are set as destinations. In nearly all networks we noticed that the selected nodes for betweenness and closenness were equivalent, therefore the Nextrout generated networks would have the same set of nodes and edges (hence the measured properties would be equivalent), so we find it enough to show the results for networks generated using only degree and betweenness centrality.

As we have already shown results obtained for the networks with OD points selected based on the degree, we now present the network properties obtained based on the betweenness centrality. Notice that despite the absolute number of destinations being the same, they still differ in terms of location, which will impact the final networks generated using our pipeline. The final betweenness properties are shown in Figures S5 and S6.

Supplementary note 4: Traffic distribution

We also show how traffic is distributed along the network. We measure traffic on edges (T_e) by setting the same origins and destination nodes used on the first step of our extraction pipeline, and running the discrete DMK-dynamics with $\beta = 1.5$. Fig. S6 shows that with this setting, both extracted and real networks tend to distribute traffic towards the more central edges, which is usually observed in real transportation systems, where stations with more connections register higher flow of passengers.

Supplementary note 5: the New York subway and some limitations of our approach

In order to recover the entire structure of the New York subway system, given its size and complexity, we first selected multiple sources (nodes with smallest degree) with one randomly selected sink among the nodes with highest degree. This strategy fails as shown in Fig. S8, where the generated topology does not show clear similarity with the original network. This evidences a limitation of our approach for selecting sources and sinks in more complex, bigger structures.

The original topology contains multiple lines that can be individually seen as an independent network itself, generating loops when superimposed. As already presented in the main text, each obtained network shows a higher similarity with the correspondent original line. A comparison of the superimposed lines is shown in Figure S9. This strategy is particularly suited for understanding more complex structures, specially for loop recovery, and it can be further explored in future work.

Supplementary note 6: the French railway system and network evolution

We show in Fig. S10 how the French railways changed in a period of around 40 years. It justifies the usage of a network from 1850 in our experiments, where the topology does not yet contain multiple loops and it is in its initial stage.

Supplementary References

1. Kujala, R., Weckström, C., Darst, R. K., Mladenović, M. N., Saramäki, J. *A collection of public transport network data sets for 25 cities*. *Scientific data*, 5:1–14, 2018. DOI: 10.5281/zenodo.3727274

2. Litvine, A., Séguy, I., Thévenin, T., et al. *French historical GIS, 1700-2020. Administrative units, populations, transports, economy*. Forthcoming, 2024. DOI: 10.5281/zenodo.3727274

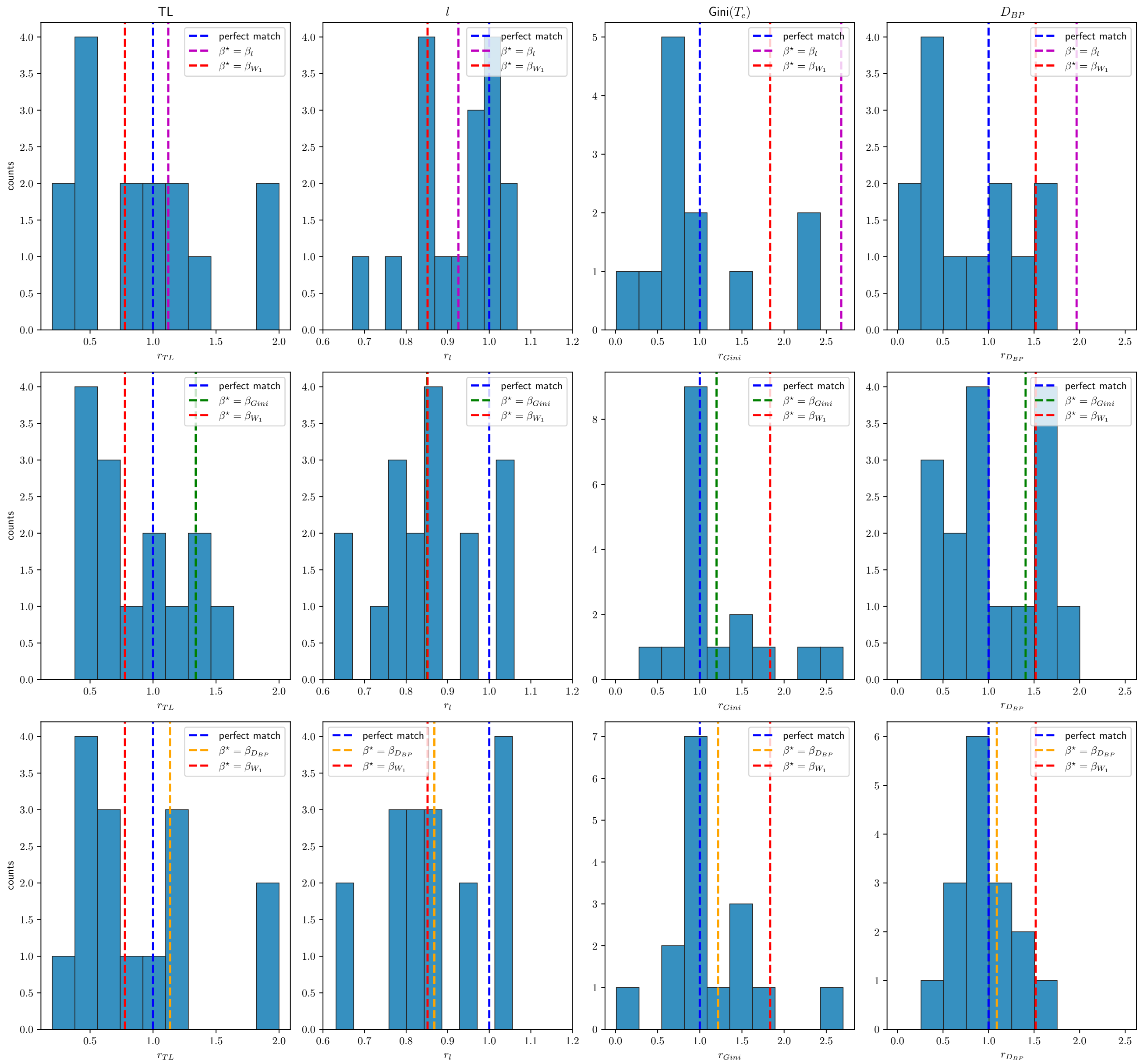

Figure S2. Metric ratios for the path length l, Gini and density of branching points. We measure the ratio r_p across the three metrics, highlighting how the average of $\beta^\star = \beta_{Wass}$ - given by the red lines - changes given other optimal β, and how it deviates from the '*perfect match*', i.e, when $r_p = 1$.

Figure 1. $\beta^{\star} = \beta_{Gini} = 1.5$

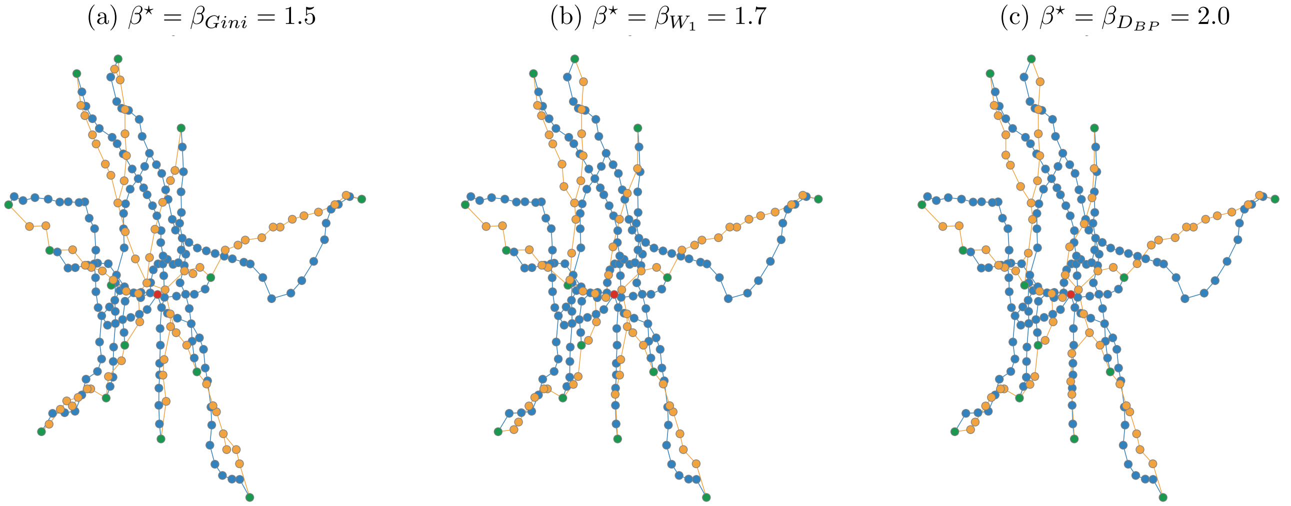

Figure S3. Optimal networks for the subway network of Berlin. In (a) the optimal β is that given by the *Gini*, which tends to favor the networks with more branches and wider coverage to distribute the traffic; in (b) we show the optimal network as selected by the Wasserstein measure, whilst in (c) we show the one given by the density of branching points, which tends to favor networks with fewer branches, so higher values of β.

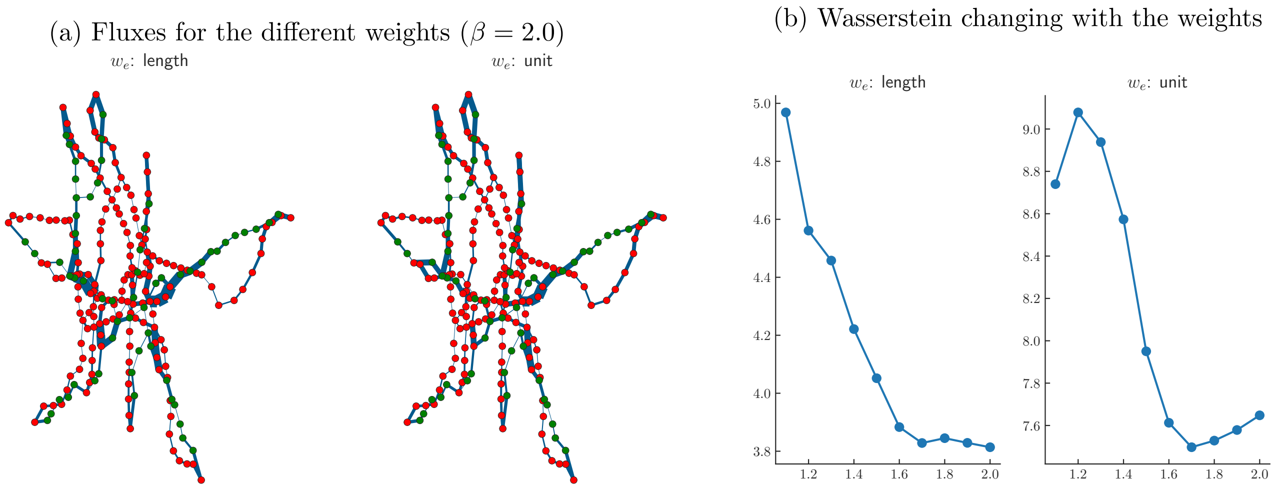

Figure S4. Selecting weights for the Wasserstein similarity measure. (a) Given the source graph (red nodes, real network) and the sink graph (green nodes, extracted network), we observe how the optimal flux distributes along the union graph. The euclidean length (left) distributes the flux along the more central nodes, thus capturing more realistic scenarios, whilst unitary weights distribute more equally along the network. We then compare in (b) how the Wasserstein measure changes across different values of β. We design the Wasserstein measure to capture more realistic scenarios, thus for smaller values of β, the networks are expected to have more redundant nodes, so we expect this measure to be higher, and the opposite as β increases. This pattern seems to be better captured by the euclidean weights.

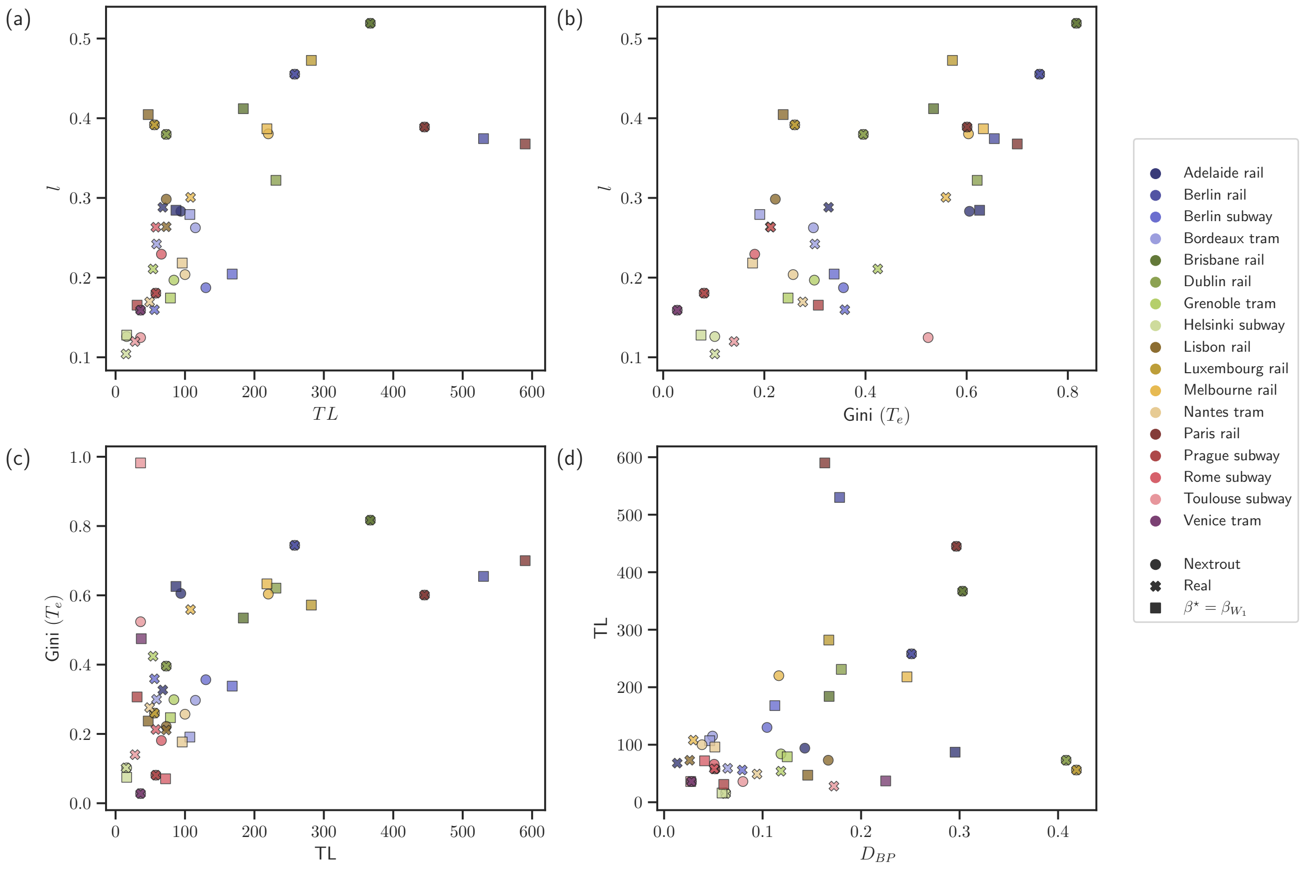

Figure S5. Measures for the studied networks. Each dataset is assigned to a different color, market shapes distinguish real and simulated networks. The ladder are further distinguished based on the one generated via Nextrout (having betweenness as the destinations selection criteria) that gives the closest point in terms of the metrics plotted in the figure (circle) or the one corresponding to the best Wasserstein measure (square). (a) We measure Cost (TL) for both simulated and real networks, plotted against the total path length l. (b) Gini coefficient as a measure of traffic distribution, versus the total path length. (c) Traffic distribution in terms of the Cost. (d) Density of bifurcations plotted against the cost.

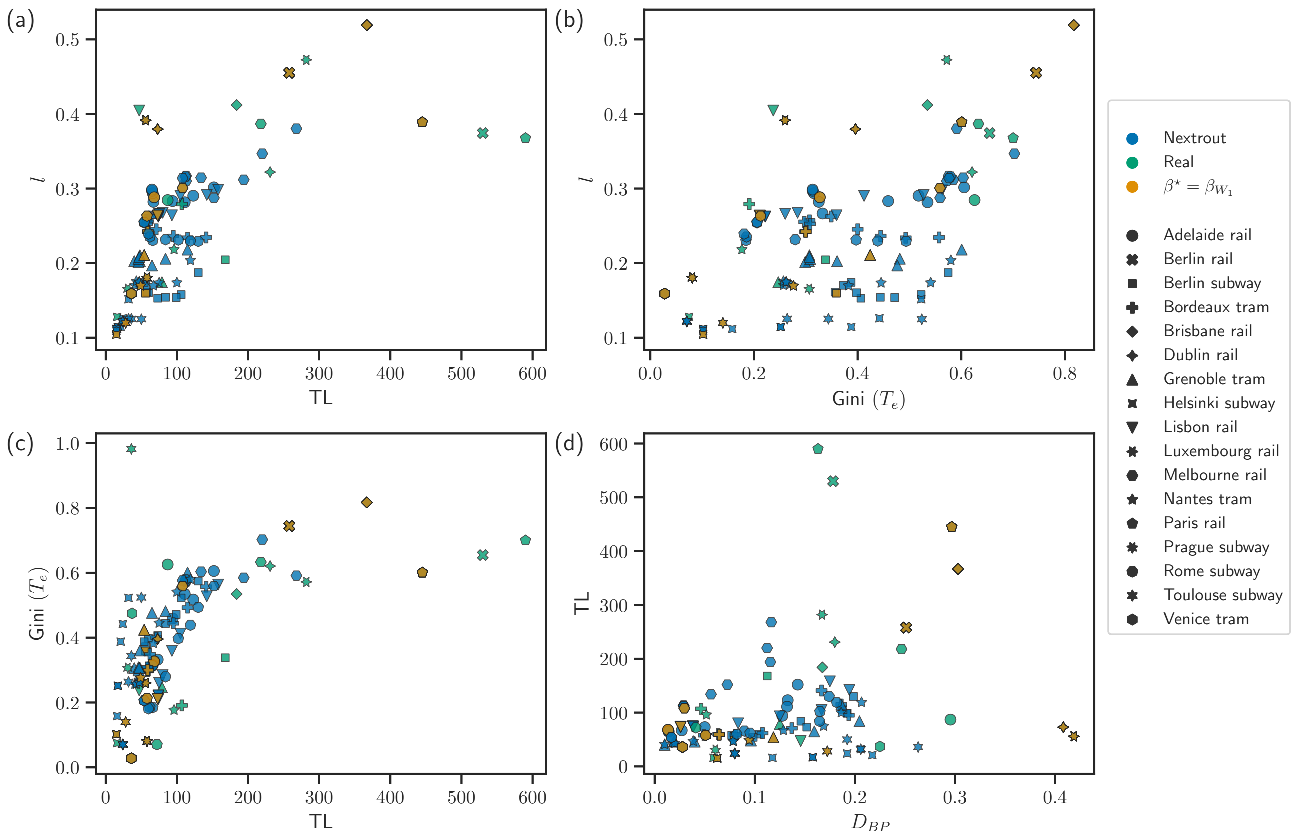

Figure S6. Comparison of simulated networks using the betweenness criteria and the one from observed data. We show the values of the main transportation properties investigated in this work for real and simulated networks. Simulated networks cover a wider range of properties' values, thus allowing in particular to select network that have lower or comparable values of these properties than those observed in the corresponding real networks.

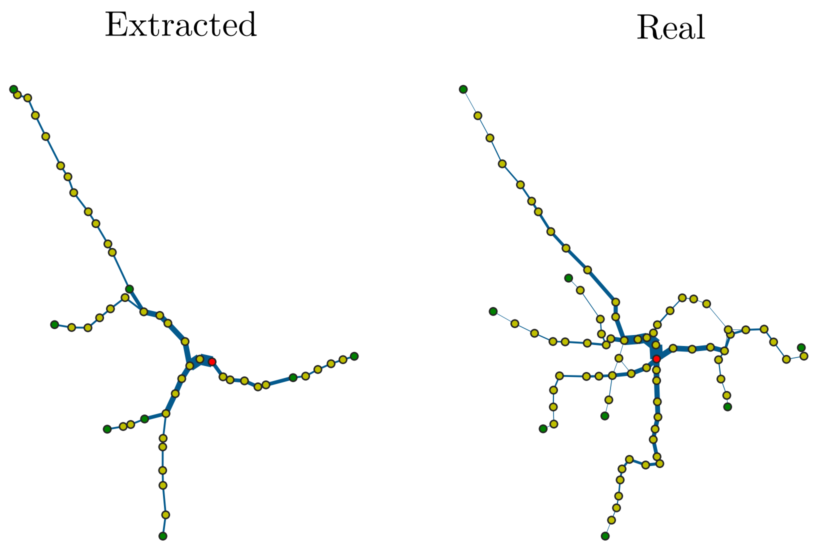

Figure S7. Traffic in extracted and real networks. Using the same set of origins and destinations we observe a higher distribution of traffic towards more central edges in both cases.

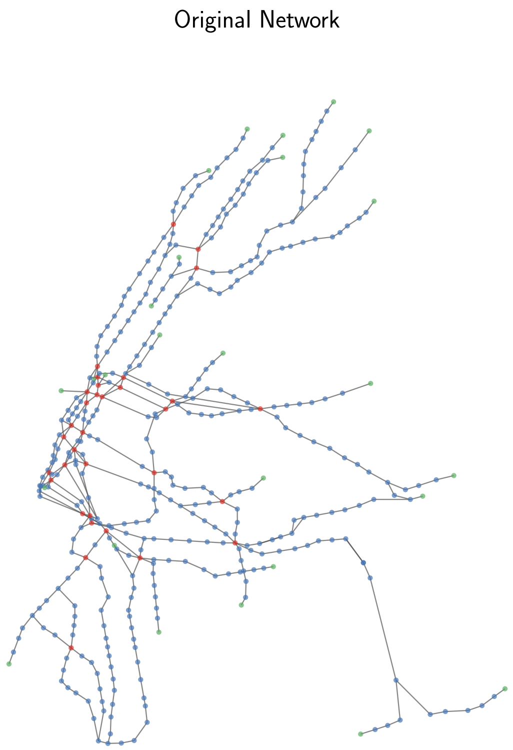

Figure S8. Left: Original New York subway network highlighting nodes with lower (green) and higher degree (red). We select one node from the higher degree ones as a sink. On the right we show the simulated network obtained with $\beta = 1.9$. Notice that despite some branches cover similar areas, such as the north-left branch, the similarity along the network is not very clear, which evidences a limitation of our approach for selecting a single sink in larger structures.

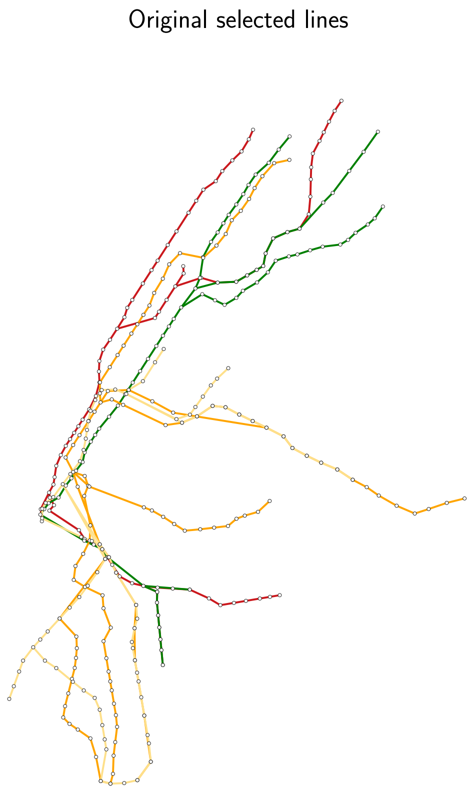

Figure S9. Overlapping selected main lines from the New York subway. We have already shown in the main text the network properties for each individual line. We now build the union of these lines, noticing that this strategy might lead to the appearance of a few loops.

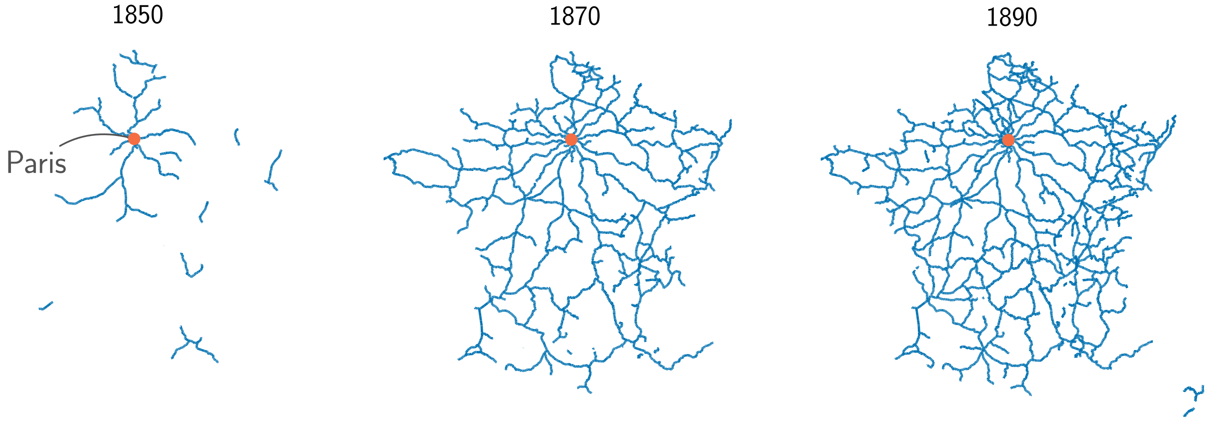

Figure S10. Evolution of the French railway network over time. We show how the network topology changes in a range of 40 years along the nineteenth century, from the selected as an example of network evolution in the main text (1850), until the emergence of multiple loops around 40 years later[2].
